# Retroperitoneal shwannoma: A case report

**DOI:** 10.1016/j.amsu.2021.102785

**Published:** 2021-09-08

**Authors:** mohamed Amine Lamris, Othmane El Yamine, Saad Rifki El Jay, Amal hajri, Rachid boufettal, Driss erreguibi, Farid Chehab

**Affiliations:** a Surgical Department of Cancerology and Liver Transplantation University Hospital Center, Casablanca, Morocco; b Faculty of Medecine and Pharmacy, Hassan II University, Casablanca, Morocco

**Keywords:** Schwannomas, Retroperitoneal, Schwann cells, Case report

## Abstract

**Introduction:**

Schwannomas are tumors that arise from Schwann cells of the peripheral nerve sheath and rarely occur in the retroperitoneum (3% of all schwannomas). Patients are usually asymptomatic or have nonspecific symptoms, making accurate preoperative diagnosis difficult. Schwannomas are usually benign, but infrequently undergo malignant transformation. Herein, we report a case of retroperitoneal schwannoma and review the relevant literature.

**Presentation of case:**

A 25-year-old woman presented to our department with a 2-year history of abdominal pain that was localized in the right flank without radiation, constipation/diarrhea or externalized digestive hemorrhage. On physical examination, we found a painless palpable mass in the right hypochondrium extending to the right iliac fossa, measuring approximately 10 cm. The MRI and CT scan showed the presence of a large intra-abdominal oval formation in the right para-umbilical region. It was well limited, measuring 110*69mm with discrete irregular contours, thickened wall and heterogeneous content mostly fluid. They also showed the presence of a cystic formation in the right ovary measuring 84*52mm and extending over 76mm. The procedure consisted of resection of the retroperitoneal solid cystic mass, right ovariectomy and drainage of the right parietal-colic gutter by Salem sump tube. A laparotomy with a median incision above and below the umbilicus was performed. After the resection, the specimens were sent for anatomopathological examination which concluded that the retroperitoneal mass was a schwannoma and the ovarian mass was a serous cystadenoma.

**Discussion:**

Retroperitoneal schwannomas are rare tumors and a pre-operative diagnosis is often difficult. The diagnosis is most often fortuitous and late, given the latency of the tumor's evolution, and the definitive diagnosis is based on histopathologic examination. Herein we presented a case of retroperitoneal schwannoma and studied the features of this phenomenon on the basis of the literature.

**Conclusion:**

Retroperitoneal schwannomas are rare. The diagnosis is often late at the stage of a large tumor. Radiologic findings are usually nondiagnostic. The treatment of choice is complete surgical excision. Prognosis is good but because of the risk of recurrence and malignant transformation, further follow-up is necessary.

## Highlights

• Schwannomas are tumors that arise from Schwann cells of the peripheral nerve sheath.
• Patients are usually asymptomatic or have nonspecific symptoms.
• The treatment of choice is complete surgical excision.
• Prognosis is good but further follow up is necessary to avoid recurrence and malignant transformation.

## 1 Introduction

**Fig. 1:**
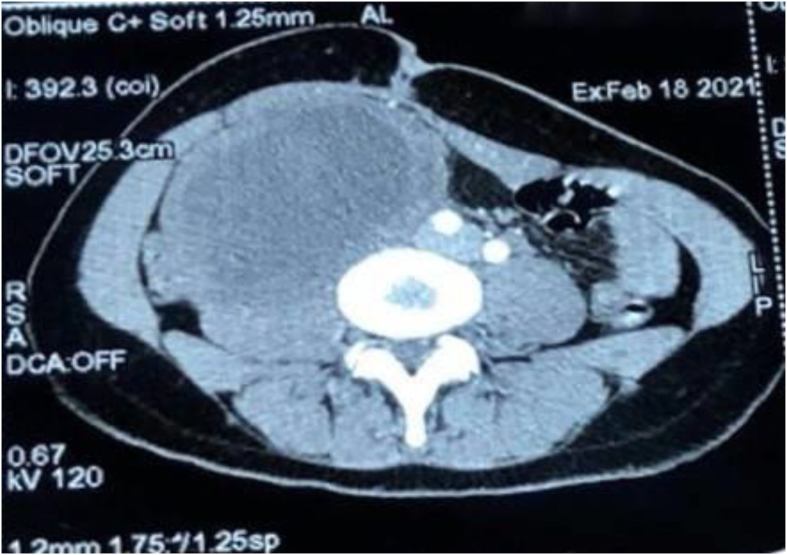
Abdominal CT scan showing the retroperitoneal Schwannoma.

**Fig. 2:**
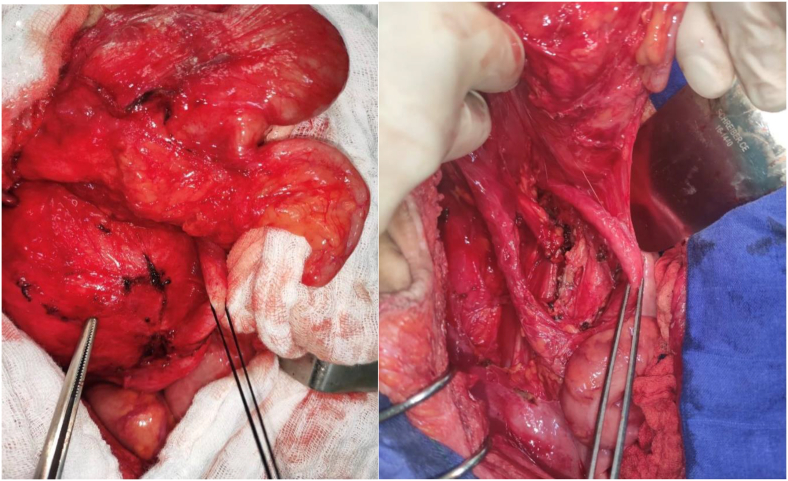
Intraoperative images of the resection of the retroperitoneal mass.

**Fig. 3:**
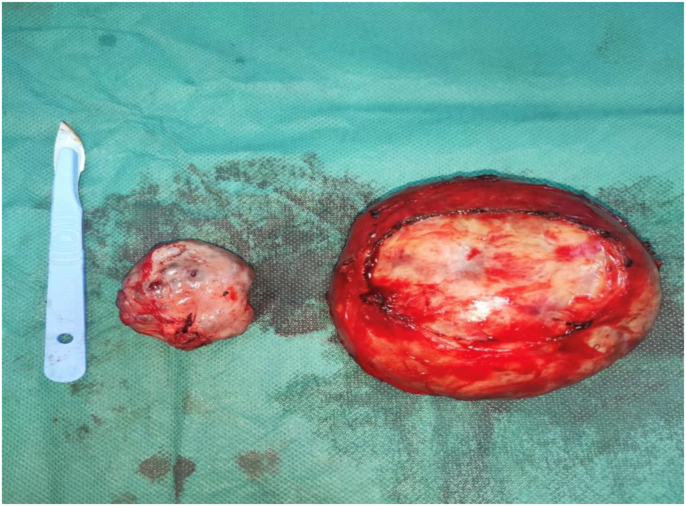
Image of the resection specimens.

Schwannomas (neurilemmomas) are soft tissue tumors that arise from Schwann cells of the peripheral nerve sheath. They usually affect the head, neck, and the flexor surfaces of the extremities. However, schwannomas may appear in the posterior mediastinum, and more rarely in the retroperitoneum, accounting for approximately 3% of all schwannomas [1]. Patients usually do not suffer from any symptoms, or exhibit nonspecific symptoms, such as abdominal pain, abdominal discomfort, constipation, and deep vein thrombosis. The lack of specific symptoms sometimes makes it difficult to accurately diagnose pre-operatively. Schwannomas are usually benign, but infrequently undergo malignant transformation. Herein, we report a case of retroperitoneal schwannoma and review the relevant literature. (Fig. 1)(see Fig. 2, Fig. 3)

## 2 Observation

A 25-year-old woman presented to our department with a 2-year history of abdominal pain that was localized in the right flank without radiation, constipation/diarrhea or externalized digestive hemorrhage. The patient was operated for a cyst of the right ovary (cystectomy) 7 years prior to the admission and for a biopsy of a retroperitoneal mass, which came back negative, 1 year prior to the admission. On physical examination, we found a painless palpable mass in the right hypochondrium extending to the right iliac fossa, measuring approximately 10 cm. On digital rectal examination, there was a bulge on the anterior wall of the rectum 6 cm from the anal margin.

The abdominal ultrasound and CT scan (Fig. 1) showed the presence of a large intra-abdominal oval formation in the right para-umbilical region. It was well limited, measuring 110*69mm with discrete irregular contours, thickened wall and heterogeneous content mostly fluid. Its wall was heterogeneously enhanced after injection of contrast medium. The tumor displaces the homolateral ureter anteriorly and medially, comes into intimate contact with the psoas muscle and the rectus abdominis muscle, and displaces the inferior vena cava and the iliac vessels. The CT scan also showed the presence of a cystic formation in the right ovary measuring 84*52mm and extending over 76mm pushing back the rectum on the homolateral side.

Pelvic MRI also showed the presence of a right paraumbilical intraperitoneal formation, roughly oval, heterogeneous in content and thick-walled, with intermediate signal on T1, hypersignal on T2, and hyposignal on diffusion, enhanced after injection of gadolinium. Another formation was visualized in the right ovary, pushing back the uterus towards the left, and reaching the contact of the rectum, with a hyposignal on T1 and diffusion, hypersignal on T2, and with high ADC, enhanced after injection of gadolinium.

A Scintigraphy and a CA-125 tumor marker dosage were performed and came back normal.

The procedure consisted of resection of the retroperitoneal solid cystic mass, right ovariectomy and drainage of the right parietal-colic gutter by Salem sump tube. A laparotomy with a median incision above and below the umbilicus was performed. On exploration, the presence of parietal-epiploic and parietal-intestinal adhesions was noted, which required adhesiolysis. After right colic-parietal detachment and lowering of the right colonic angle, a retroperitoneal solid-cystic mass of 20cm long was found, it was well limited, with regular contours, pushing back the right ureter anteriorly and medially, the inferior vena cava posteriorly, the right iliac vessels inferiorly and medially and the duodenum upwards. We also found a right cystic ovarian mass measuring 10cm in long axis. After locating the right ureter, the retroperitoneal mass was dissected from the inferior vena cava and the right iliac vessels before resection. A right ovariectomy was then performed after vascular control with LigaSure forceps.

Finally, a drainage of the right parieto-colic gutter was performed by Salem's sump tube after a peritoneal toilet with saline. The specimens were sent for anatomopathological examination which concluded that the retroperitoneal mass was a schwannoma and the ovarian mass was a serous cystadenoma.

This case report has been reported in line with the SCARE Criteria [2].

## 3 Discussion

Schwannomas (neurilemmomas) are soft tissue tumors that arise from Schwann cells of the peripheral nerve sheath and predominantly occur in women aged between their 2nd and 5th decades [3]. Only a small number of schwannomas are found in the retroperitoneum, they account for up to 6% of all primary retroperitoneal tumors and are usually located in the paravertebral or presacral space [1,3]. The diagnosis is most often fortuitous and late, given the latency of the tumor's evolution. Patients with retroperitoneal schwannomas (RS) usually do not suffer from any symptoms, or exhibit nonspecific symptoms, such as abdominal pain, abdominal discomfort, constipation, and deep vein thrombosis [4]. The lack of specific symptoms makes the diagnosis of retroperitoneal schwannomas a diagnosis of exclusion. Imaging modalities for RS include abdominal ultrasound, computed tomography (CT), and magnetic resonance imaging (MRI). Abdominal ultrasound is a rapid, inexpensive, and effective tool for initial diagnostic orientation. It provides information on the cystic and solid nature of the schwannoma [5]. The abdominal CT scan generally shows a well limited solid tumor with a cystic component. It provides the same information as the MRI but the MRI is accepted as the imaging method of choice for diagnosis of most soft-tissue tumors because of its higher diagnostic predictability compared with ultrasound or CT [3,5]. Schwannomas are characterized by a hyposignal on T1 and a heterogeneous hypersignal on T2 [6]. However, MRI cannot distinguish between benign and malignant lesions. Some signs have been reported as signs of malignancy such as irregular contours, heterogeneity and large size (>5 cm) [7]. Definitive diagnosis and determination of the nature of schwannoma are based on a histological examination. Histologically, schwannomas are distinguished by the presence of areas of high and low cellularity called Antoni A and Antoni B tissue, respectively [8]. Preoperative CT- or ultrasound-guided biopsy is not recommended by most authors because of the difficulties of interpretation, and may be associated with the risk of bleeding, infection, and tumor seeding. Therefore, a definitive diagnosis of schwannoma should be made on postoperative histopathology [5,9]. Schwannomas are not sensitive to radiotherapy and chemotherapy, the only valid treatment is complete surgical excision [10]. However, controversy exists over the necessity of negative soft-tissue margins especially when adjacent tissue or viscera need to be sacrificed. Although some advocate complete excision because of the risk of recurrence and malignancy that cannot be excluded preoperatively, others believe that simple enucleation or partial excision is sufficient [1,3,11]. The prognosis of benign schwannomas is good and the malignant transformation is exceptional. The most frequent complication is recurrence, probably due to incomplete excision, which is reported in 5–10% cases [5,12]. Therefore, these patients require long-term follow-up that includes a clinical examination and CT scan at 6 and 12 months after surgery, and then annually for five years [5].

## 4 Conclusion

Retroperitoneal schwannomas are rare. The diagnosis is often late at the stage of a large tumor. Radiologic findings are usually nondiagnostic. The treatment of choice is complete surgical excision. Prognosis is good but because of the risk of recurrence and malignant transformation, further follow-up is necessary.

## Author contribution

All the authors contributed to study concept, data analysis and writing the paper.

## Guarantor

Dr Lamris Mohamed Amine and Dr El Yamine Othmane are the guarantors for this study.

## Funding

This research did not receive any specific grant from funding agencies in the public, commercial, or not-for-profit sectors.

## Ethical approval

This case report is exempt from ethical approval at our institution.

## Consent

Written informed consent was obtained from the patient for publication of this case report and accompanying images. A copy of the written consent is available for review by the Editor-in-Chief of this journal on request.

## Registration of research studies

The datasets in this article are available in the repository of the general surgery database, CHU Ibn Rochd, upon request, from the corresponding author.

## Provenance and peer review

Not commissioned, externally peer reviewed.

## Declaration of competing interest

The authors report no declarations of interest.
